# Ketorolac tromethamine pretreatment suppresses sufentanil-induced cough during general anesthesia induction: a prospective randomized controlled trial

**DOI:** 10.1186/s12871-020-01124-5

**Published:** 2020-08-17

**Authors:** Zhen Tian, Bei Hu, Min Miao, Lulu Zhang, Lin Wang, Bin Chen

**Affiliations:** 1 Department of Anesthesiology, Suqian People’s Hospital of Nanjing Drum-tower Hospital Group, Suqian, 223800 China; 2 Department of Anesthesiology, The Affiliated Suqian Hospital of Xuzhou Medical University, Suqian, 223800 China

**Keywords:** Ketorolac tromethamine, Sufentanil, Cough response, General anesthesia

## Abstract

**Background:**

To observe the effect of pretreatment with ketorolac tromethamine on sufentanil-induced cough in general anesthesia patients.

**Methods:**

A total of 102 patients were screened, and 90 patients were scheduled for elective surgery under general anesthesia. The 90 patients were randomly divided into two groups: the control group (C group) and the observation group (KT group). Five minutes before anesthesia induction, the observation group was given ketorolac tromethamine 0.5 mg/kg intravenously within 3 s, while the control group was given the same amount of normal saline intravenously. All patients were given a sufentanil bolus of 0.5 μg/kg (within 3 s) intravenously. One minute later, propofol 2.5 mg/kg and vecuronium 0.15 mg/kg were injected intravenously, and endotracheal intubation was guided by laryngoscopy. The number of coughs that occurred within 1 min after sufentanil injection was recorded. The mean arterial pressure (MAP), heart rate (HR) and pulse oxygen saturation (SpO_2_) were recorded at T0 (immediately before pretreatment), T1 (5 min after pretreatment), T2 (before intubation), T3 (1 min after intubation) and T4 (5 min after intubation). The incidence of adverse reactions, including nausea and vomiting, dizziness, drowsiness, delay of recovery, restlessness in the recovery period, respiratory depression and postoperative incision pain, was analyzed.

**Results:**

Within 1 min after sufentanil injection, the incidence and severity of cough in the KT group was significantly lower than that in the C group (*P* <  0.05). At T0, T1, T2, T3 and T4, there were no significant differences in MAP, HR and SpO2 between the two groups (*P* >  0.05). There was no significant difference in the dosage of sufentanil, propofol, remifentanil and vecuronium, the incidence of nausea and vomiting, the delay of recovery, dizziness, drowsiness or respiratory depression between the two groups (*P* >  0.05). However, the incidence of restlessness and the number of patients with VAS scores > 3 in the KT group were significantly lower than those in the C group (*P* <  0.05).

**Conclusion:**

Pretreatment with intravenous ketorolac tromethamine can significantly reduce the incidence of sufentanil-induced cough during induction of general anesthesia, which can also significantly reduce postoperative incision pain and restlessness during the recovery period.

**Trial registration:**

Chinese Clinical Trial Registry (registration number# ChiCTR2000030287; date of registration: 27/02/2020).

## Background

Sufentanil, commonly used in general anesthesia for its strong analgesic properties, can produce an irritating cough during intravenous induction [1, 2]. Studies have shown that 25 to 42% of patients experience this kind of cough reaction after intravenous injection of sufentanil [2, 3]. Sometimes, the cough reaction will be explosive or spasmodic, which will cause a sharp rise in blood pressure, intracranial pressure, intraocular pressure, intrapulmonary pressure and abdominal pressure. Therefore, for patients with hypertension, intracranial hypertension, pulmonary bullae, hemangioma and high intra-abdominal pressure, the risks of anesthesia induction are significantly increased when using sufentanil [4].

Various agents, such as remifentanil, dexmedetomidine, magnesium sulfate, lidocaine, and dezocine, have been used to suppress cough during the induction of general anesthesia [5, 6]. However, their clinical application was somewhat limited due to their potential additional side effects, including sharp decreases in heart rate and blood pressure, myocardial inhibition, respiratory depression, long onset time, long duration and low effective rate [7, 8]. Therefore, it is a challenge for anesthesiologists to find a safe and effective drug with fewer adverse reactions to inhibit the sufentanil-induced cough response during the induction of general anesthesia.

Aspirin is a common nonsteroidal anti-inflammatory and analgesic drug. Alexander T et al. reported that a moderate dose of aspirin (500 mg/day) could reduce angiotensin converting enzyme inhibitor-induced cough [9]. Ketorolac tromethamine is a new nonsteroidal anti-inflammatory and analgesic drug that is widely used in the short-term treatment of acute moderate and severe pain, including postoperative incision pain [10, 11]. At present, the clinical effect and safety of ketorolac tromethamine for suppressing sufentanil-induced cough during the induction of general anesthesia remain unclear.

The purpose of this study was to investigate the effectiveness of ketorolac tromethamine for treating the sufentanil-induced cough response by intravenous injection prior to general anesthesia induction.

## Materials and methods

This study was approved by the Institutional Research Ethics Committee of the Suqian People’s Hospital of Nanjing Drum-Tower Hospital Group, Suqian, Jiangsu, China. The trial was registered in the Chinese Clinical Trial Registry (No. Chi CTR2000030287). Written informed consent was obtained from each patient following the principles of the Helsinki Declaration. And This study is adhered to CONSORT guidelines.

A total of 102 adult patients with ASA physical status I or II, aged 18–65 years, weighing 45 to 89 kg, with BMIs between 18.0 and 30.0 kg/m^2^, were enrolled in this study. The patients were scheduled for elective surgery under general anesthesia in Suqian People’s Hospital of Nanjing Drum-Tower Hospital Group from February 2020 to March 2020. The exclusion criteria were a history of asthma, chronic cough, and upper respiratory tract infection within 2 weeks. Patients with a history of peptic ulcer or bleeding, heart disease, aneurysm, liver disease, kidney disease, or participation in other clinical trials or who the researchers considered inappropriate to participate in this experiment were also excluded. Ninety patients were randomly divided into two groups via a computer-generated random number list: the control group (C Group, *n* = 45) and the observation group (KT Group, *n* = 45).

No patient received premedication in this study. In the operating room, noninvasive blood pressure (NBP), pulse oxygen saturation (SpO_2_), and electrocardiograms (ECGs) were routinely monitored. Patients were cannulated through the median cubital vein of the forearm with a 20G venous trocar needle. 5 min before general anesthesia induction, KT group patients were given ketorolac tromethamine 0.5 mg/kg (diluted to 5 ml using normal saline) intravenously within 3 s, while those in the C group were given 5 ml of normal saline only. The ketorolac tromethamine or normal saline was prepared by a nurse anesthetist and administered by an experienced anesthesiologist who was blind to the procedure. All patients were given 100% oxygen via a face mask with an oxygen flow rate of 5 L/min for 3 min. General anesthesia was induced with a bolus of sufentanil 0.5 μg/kg administered within 3 s intravenously, and 1 min later, propofol 2.5 mg/kg and vecuronium 0.15 mg/kg were infused sequentially. Endotracheal intubation was performed using a Macintosh laryngoscope. The depth of general anesthesia was maintained under propofol 5 mg/kg/h, remifentanil 10 μg/kg/h and vecuronium 0.05 mg/kg/h and was adjusted based on the vital signs of the patients.

The frequency of cough within 1 min following sufentanil injection was recorded, and the severity was graded depending on the cough frequency (mild, 1–2; moderate, 3–4; severe, ≥ 5, [12, 13]).

The mean arterial pressure (MAP), heart rate (HR) and S_P_O_2_ were recorded at the following time-points: T0, before pretreatment of ketorolac tromethamine or normal saline, i.e., the baseline value; T1, 5 min after pretreatment; T2, before intubation; T3, 1 min after intubation; and T4, 5 min after intubation.

The incidence of adverse reactions, including nausea and vomiting, dizziness, drowsiness, delay of recovery, restlessness in the recovery period and respiratory depression, was analyzed. In brief, the adverse reactions of the two groups were evaluated by professional anesthesiologists according to the following unified criteria: because nausea and dizziness are subjective assessments, for the evaluation of nausea and dizziness, the patients (who had recovered from the anesthesia) were questioned by the anesthesiologist and were recorded as having nausea and/or dizziness if the answer was “Yes”. The vomiting judgment was performed according to the patient’s self-report and the anesthesiologist’s observation. Drowsiness was defined as sleeping again within 120 s of waking up without external interference. Recovery delay referred to the patient’s consciousness not being recovered and the patient being unable to make correct responses to external stimulation and language instruction 120 min after general anesthesia. Restlessness in the recovery period was defined as the patients mood being more excited in the recovery period, even crying and being restless, and the patients making strong movements that could not be comforted. Respiratory inhibition referred to respiratory arrest time greater than 15 s or S_P_O_2_ less than 90% for more than 15 s when patients without oxygen inhaled during the recovery period. The postoperative incision pain of the patients was evaluated by the VAS scoring method, with a score of 0–10. The number of patients with VAS scores > 3 was recorded.

### Sample size determination

In our preliminary study, the incidence of cough elicited by 0.5 μg/kg sufentanil infused within 3 s was 31.8% (7/22), which was reduced to 4.5% (1/22) when ketorolac tromethamine pretreatment was performed. To achieve 80% statistical power with α = 0.05, each group would require no less than 33 cases. Considering that there may be a dropout rate of 20%, we recruited 51 patients for each group to allow for missing data.

### Statistical analysis

SPSS 22.0 software (IBM Corp, Armonk, NY, USA) was used for statistical analysis. The presented data were evaluated for normal distributions by the Kolmogorov–Smirnov test. Measurement data are presented as the mean ± standard deviation, and Student’s t test was used to assess the differences between two groups. The differences in ranked data were analyzed by the Mann–Whitney U test. The chi-square test or Fisher’s exact test was adopted to assess the difference in categorical data presented as absolute or relative effect sizes. A *P*-value < 0.05 was considered significant.

## Results

**Fig. 1:**
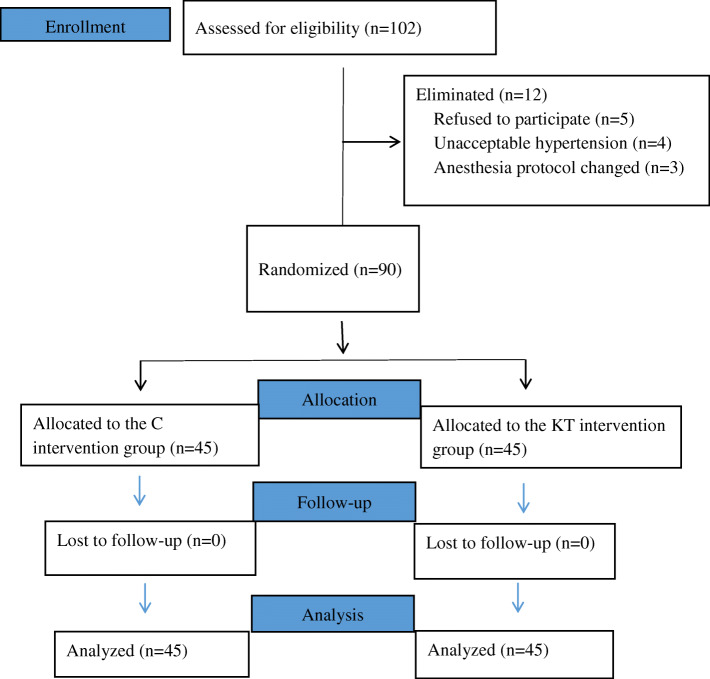
Flow chart of patients participating in this study

Among 102 patients, 5 patients refused to participate in the study, 4 patients had a history of hypertension, and 3 patients underwent a change in anesthesia protocol (Fig. 1); therefore, 90 patients were enrolled for further study. There were no significant differences in sex, age, weight, BMI, ASA physical status or anesthesia time between the two groups (*P* >  0.05) (Table 1).

**Table 1:** Demographic data and basic clinical characteristics of the patients in the two groups

	C Group	KT Group	*P* value
Sex (male/female)	19/26	21/24	> 0.05
Age (year)	47.8 ± 8.9	48.1 ± 9.1	> 0.05
ASA (I/II)	28/17	30/15	> 0.05
Weight (kg)	69.8 ± 10.2	69.4 ± 9.9	> 0.05
BMI (kg/m^2^)	24.6 ± 3.4	24.9 ± 3.1	> 0.05
Anesthesia time (min)	95.9 ± 20.8	97.0 ± 21.0	> 0.05

Values are expressed as the mean ± standard deviation

The incidence and severity of cough within 1 min after sufentanil injection in the KT group was significantly lower than that in the C group (*P* <  0.05) (Table 2).

**Table 2:** Incidence and severity of cough in the two groups

Groups	Incidence of cough (n(%))	Severity of cough (n(%))			
		None	Mild	Moderate	Severe
C group	14(31.1)	31(68.9)	3(6.7)	11(24.4)	0(0.0)
KT group	3(6.7)	42(93.3)	2(4.4)	1(2.2)	0(0.0)
*P* value	< 0.05	< 0.05			

There were no significant differences in MAP, HR or S_P_O_2_ between the two groups at T0, T1, T2, T3 and T4 (*P* >  0.05) (Table 3).

**Table 3:** Comparison of MAP, HR and SpO2 values at different time points

Groups		T0	T1	T2	T3	T4
C group	MAP (mmHg)	94.7 ± 11.2	94.0 ± 9.8	84.3 ± 10.5	90.9 ± 10.9	82.7 ± 9.5
	HR (bpm)	76.3 ± 7.5	75.8 ± 6.2	69.2 ± 7.4	79.1 ± 7.1	68.7 ± 6.4
	SpO_2_ (%)	98.1 ± 1.2	97.8 ± 0.9	99.0 ± 0.7	99.0 ± 0.8	99.0 ± 1.0
KT group	MAP (mmHg)	93.9 ± 11.5	93.4 ± 9.6	83.9 ± 10.2	89.2 ± 10.3	81.9 ± 9.3
	HR (bpm)	75.9 ± 7.3	75.2 ± 6.0	68.9 ± 7.6	78.8 ± 7.0	68.4 ± 6.6
	SpO_2_ (%)	97.9 ± 1.0	97.7 ± 0.8	98.8 ± 0.7	99.0 ± 1.0	99.2 ± 0.5

Values are expressed as the mean ± standard deviation

There was no significant difference in the dosage of sufentanil, propofol, remifentanil and vecuronium between the two groups (*P* >  0.05) (Table 4).

**Table 4:** Comparison of anesthetic dosages between the two groups

Groups	C group	KT group	*P* value
Sufentanil (μg)	39.2 ± 8.0	38.7 ± 7.8	> 0.05
Propofol (mg)	640.4 ± 85.4	629.3 ± 84.5	> 0.05
Vecuronium (mg)	17.0 ± 3.8	16.5 ± 3.6	> 0.05
Remifentanil (mg)	1.2 ± 0.3	1.1 ± 0.2	> 0.05

Values are expressed as the mean ± standard deviation

The incidences of restlessness and the number of patients with VAS scores > 3 in the anesthesia recovery period in the KT group were significantly lower than those in the C group (*P* <  0.05). There were no significant differences in the incidences of nausea and vomiting, delay of recovery, dizziness, drowsiness or respiratory depression between the two groups (*P* >  0.05) (Table 5).

**Table 5:** Comparison of adverse reactions during the recovery period (n(%))

Groups	C group	KT group	*P* value
Nausea and vomiting	3 (6.7)	4 (8.9)	> 0.05
Delay of recovery	1 (2.2)	2 (4.4)	> 0.05
Dizziness	3 (6.7)	3 (6.7)	> 0.05
Drowsiness	2 (4.4)	3 (6.7)	> 0.05
Restlessness in the recovery period	7 (15.6)	1 (2.2)	< 0.05
Depressed respiration	0 (0.0)	1 (2.2)	> 0.05
VAS score > 3	8 (17.8)	1 (2.2)	< 0.05

## Discussion

At present, sufentanil, fentanyl and other opioid analgesics injected intravenously during the induction period of clinical anesthesia show strong analgesic effects and little influence on hemodynamic indexes [14, 15], and they can effectively inhibit tracheal intubation responses (such as increased heart rate and increased blood pressure) [16, 17]. However, it is easy to cause coughing reactions of different degrees within 1 min of intravenous injection [18]. For patients with hypertension, pulmonary bullae, hemangioma and intracranial hypertension, this may have severe consequences.

The various incidence rates among different studies might be due to the different doses of sufentanil used and differences in concentration, administration rate, race and age [19]. In a study by Agarwal et al., sufentanil 0.3 μg/kg injected over 5 s elicited cough in 15.8% of patients, while in another study by Li et al., the incidence of cough was 37% after the injection of sufentanil 0.5 μg/kg within 3 s, and with a high dose of sufentanil (1 μg/kg), the incidence of sufentanil-induced cough could be up to 45.8% [20, 21]. In our study, we administered sufentanil 0.5 μg/kg intravenously within 3 s before the operation in the C group. Within 1 min, the incidence of sufentanil-induced cough was 31.1%, which is similar to the conclusions of relevant research.

Five minutes before anesthesia induction, intravenous ketorolac tromethamine 0.5 mg/kg pretreatment can significantly reduce the incidence of coughing reactions during the induction period in general anesthesia patients and can significantly reduce restlessness in the recovery period of patients, which increases safety.

Many studies have been performed on the mechanism of the sufentanil-induced cough response, but the relevant mechanism is still not very clear [3–5]. It may be related to sufentanil activating the C-fiber receptor of the bronchus, adapting the pulmonary stretch receptors (RARs), inducing airway hyperresponsiveness, inhibiting the efferent impulse of the sympathetic nerve, increasing the comparative advantage of the vagus nerve, and finally leading to the occurrence of the cough response [5, 6]. At present, there are few reports about ketorolac tromethamine reducing the sufentanil-induced cough response. It has been reported that intravenous injection of dezocine before anesthesia induction can inhibit the cough response induced by sufentanil or fentanyl to some extent by activating the *K* receptor and inhibiting histamine release [5]. Considering that ketorolac tromethamine is a nonsteroidal anti-inflammatory drug, the mechanism of a ketorolac tromethamine injection reducing the cough response may be related to the reduction of histamine release and other reasons [10].

Ketorolac tromethamine is a new nonsteroidal anti-inflammatory drug (NSAID). Motov S et al. found that intravenous infusion of 30 mg ketorolac tromethamine can significantly improve moderate to severe pain in emergency patients [22]. Studies by Yang HL et al. suggested that injection of ketorolac tromethamine before tracheal intubation can reduce the incidence of sore throat caused by endotracheal intubation from 71.6 to 21.1% [23]. Compared with other NSAIDs, ketorolac tromethamine has a weaker anti-inflammatory effect and stronger analgesic effect and has been widely used in the clinic [10, 24]. In this study, ketorolac tromethamine significantly reduced the number of patients with VAS scores greater than 3. Pretreatment with a ketorolac tromethamine injection also played a role in the recovery period of anesthesia, significantly reducing the postoperative incision pain of patients, and the incidence of restlessness caused by pain and other adverse stimulation naturally decreased significantly. To a certain extent, it provided patients with a comfortable medical experience and humane care.

To evaluate the possible adverse reactions and safety of ketorolac tromethamine injection pretreatment, we compared the mean arterial pressure, heart rate and blood oxygen saturation values of the two groups of patients at different time points. The results suggest that pretreatment with ketorolac tromethamine injection does not have adverse effects on the vital signs of patients. Additionally, the results showed that the incidence of nausea, delayed recovery, dizziness, drowsiness and respiratory depression was not significantly increased when ketorolac tromethamine was used in advance, making it safe for patients. However, it is undeniable that one of the main risks of NSAIDs, such as ketorolac tromethamine, is that they may affect bleeding during and after the operation by inhibiting platelet function. However, the dosage of drugs used in this study was not large and was within the scope of reasonable use in the instructions. In addition, this study has limited the research subjects; for example, patients who had a history of peptic ulcer or bleeding, liver or kidney disease, or blood system diseases before operation were excluded. Under these premises, ketorolac tromethamine will not have a significant impact on the operation or postoperative bleeding.

There are also some deficiencies in our research. First, due to the limitation of objective conditions, we have not studied the mechanism of ketorolac tromethamine in inhibition of the cough response and cannot give more reasonable inferences about the relevant mechanisms. We describe the relevant effects objectively because there is no relevant report about ketorolac tromethamine injection or other nonsteroidal drugs inhibiting sufentanil-induced cough. Second, the pretreatment dose of ketorolac tromethamine that was used (0.5 mg/kg) may not be the most appropriate dose for ketorolac tromethamine to inhibit the sufentanil-induced cough response, but it was given according to the early postoperative analgesic dose recommended in the drug instructions [10]. Third, our study was a single center study with a small sample size. To determine whether pretreatment with ketorolac tromethamine injection can reduce the sufentanil-induced cough response, we still need a large sample and multicenter study.

## Conclusion

Pretreatment with intravenous ketorolac tromethamine 0.5 mg/kg can significantly reduce the incidence and severity of sufentanil-induced cough during induction of general anesthesia, which can also significantly and safely reduce postoperative incision pain and restlessness in the recovery period. To a certain extent, it provided a comfortable medical experience and humane care, which is worth popularizing.

## Data Availability

The datasets used and/or analyzed during the current study are available from the corresponding author on reasonable request.

## Abbreviations

NBP: Noninvasive blood pressure
SpO_2_: Pulse oxygen saturation
ECG: Electrocardiogram
MAP: Mean arterial pressure
HR: Heart rate

## References

[1] Ambesh SP, Singh N, Srivastava K (2009). Fentanyl induced coughing caused life-threatening airway obstruction in a patient with arteriovenous malformation of tongue and hypopharynx. Internet J Anesthesiology.

[2] Tang Q, Qian Y, Zhang Q, Yang J, Wang Z (2010). Effects of different priming doses of propofol on fentanyl-induced cough during anesthesia induction: a preliminary randomized controlled study. Ups J Med Sci.

[3] Shen JC, Xu JG, Zhou ZQ, Liu HJ, Yang JJ (2008). Effect of equivalent doses of fentanyl, sufentanil, and remifentanil on the incidence and severity of cough in patients undergoing abdominal surgery: a prospective, randomized, double-blind study. Curr Ther Res Clin Experimental.

[4] Yoo YC, Na S, Jeong JJ, Choi EM, Moon BE, Lee JR (2011). Dose-dependent attenuation by fentanyl on cough during emergence from general anesthesia. Acta Anaesthesiol Scand.

[5] Xu Y, Zhu Y, Wang S, Ren Y, Miao C (2015). Dezocine attenuates fentanyl-induced cough in a dose-dependent manner-a randomized controlled trial. Int J Clin Exp Med.

[6] Sridharan K, Sivaramakrishnan G (2019). Comparison of fentanyl, Remifentanil, Sufentanil and Alfentanil in combination with Propofol for general anesthesia: a systematic review and meta-analysis of randomized controlled trials. Curr Clin Pharmacol.

[7] Liu XS, Xu GH, Shen QY, Zhao Q, Cheng XQ, Zhang J, Gu EW (2015). Dezocine prevents sufentanil-induced cough during general anesthesia induction: a randomized controlled trial. Pharmacol Rep.

[8] Sun S, Huang SQ (2013). Effects of pretreatment with a small dose of dexmedetomidine on sufentanil-induced cough during anesthetic induction. J Anesth.

[9] Tenenbaum A, Grossman E, Shemesh J, Fisman EZ, Nosrati I, Motro M (2000). Intermediate but not low doses of aspirin can suppress angiotensin-converting enzyme inhibitor-induced cough. Am J Hypertens.

[10] Gurunathan U, Parker SL, Maguire R, Ramdath D, Bijoor M, Wallis SC, Roberts JA (2019). Population pharmacokinetics of Periarticular ketorolac in adult patients undergoing Total hip or Total knee replacement surgery. Anesth Analg.

[11] Vadivelu N, Gowda AM, Urman RD, Jolly S, Kodumudi V, Maria M, Taylor R, Pergolizzi JV (2015). Ketorolac tromethamine - routes and clinical implications. Pain Pract.

[12] Lin W, Sun J, Fu S (2019). A small dose of remifentanil pretreatment suppresses sufentanil-induced cough during general anesthesia induction: a randomized, double-blind, placebo-controlled trial. BMC Anesthesiol.

[13] Zhou W, Zhang D, Tian S, Yang Y, Xing Z, Ma R, Zhou T, Bao T, Sun J, Zhang Z (2019). Optimal dose of pretreated-dexmedetomidine in fentanyl-induced cough suppression: a prospective randomized controlled trial. BMC Anesthesiol.

[14] Pandey CK, Raza M, Ranjan R, Lakra A, Agarwal A, Singh U, Singh RB, Singh PK (2004). Intravenous lidocaine suppresses fentanyl-induced coughing: a double-blind, prospective, randomized placebo-controlled study. Anesth Analg.

[15] Firouzian A, Emadi SA, Baradari AG, Mousavi R, Kiasari AZ (2015). Can low dose of propofol effectively suppress fentanyl-induced cough during induction of anaesthesia? A double blind randomized controlled trial. J Anaesthesiol Clin Pharmacol.

[16] Li Y, Wang B, Zhang LL, He SF, Hu XW, Wong GT, Zhang Y (2016). Dexmedetomidine combined with general anesthesia provides similar intraoperative stress response reduction when compared with a combined general and epidural anesthetic technique. Anesth Analg.

[17] Sun ZT, Yang CY, Cui Z, Zhang J, Han XP (2011). Effect of intravenous dezocine on fentanyl-induced cough during general anesthesia induction: a double-blinded, prospective, randomized, controlled trial. J Anesth.

[18] Shrestha SK, Bhattarai B, Shah RS (2012). Preemptive use of small dose fentanyl suppresses fentanyl induced cough. Kathmandu Univ Med J (KUMJ).

[19] An LJ, Gui B, Su Z, Zhang Y, Liu HL (2015). Magnesium sulfate inhibits sufentanil-induced cough during anesthetic induction. Int J Clin Exp Med.

[20] Agarwal A, Gautam S, Nath SS, Gupta D, Singh U (2007). Comparison of the incidence and severity of cough induced by sufentanil and fentanyl: a prospective, randomised, double-blind study. Anaesthesia..

[21] Li J, Li K, Sadeghian SH, Mehta U, Rajbongshi P (2016). Effects of pre-inhalation of salbutamol on cough reflex induced by Sufentanil. AER-Advances in Engineering Research.

[22] Motov S, Yasavolian M, Likourezos A, Pushkar I, Hossain R, Drapkin J, Cohen V, Filk N, Smith A, Huang F (2017). Comparison of intravenous ketorolac at three single-dose regimens for treating acute pain in the emergency department: a randomized controlled trial. Ann Emerg Med.

[23] Yang HL, Liu FC, Tsai SC, Tsay PK, Lin HT, Liu HE (2016). Ketorolac Tromethamine spray prevents Postendotracheal-intubation-induced sore throat after general anesthesia. Biomed Res Int.

[24] Almeida DR, Johnson D, Hollands H, Smallman D, Baxter S, Eng KT, Kratky V, ten Hove MW, Sharma S, El-Defrawy S (2008). Effect of prophylactic nonsteroidal antiinflammatory drugs on cystoid macular edema assessed using optical coherence tomography quantification of total macular volume after cataract surgery. J Cataract Refract Surg.

